# Control of Glycosylation-Related Genes by DNA Methylation: the Intriguing Case of the *B3GALT5* Gene and Its Distinct Promoters

**DOI:** 10.3390/biology3030484

**Published:** 2014-08-04

**Authors:** Marco Trinchera, Aida Zulueta, Anna Caretti, Fabio Dall’Olio

**Affiliations:** 1 Department of Medicine Clinical and Experimental (DMCS), University of Insubria, 21100 Varese, Italy; 2 Department of Health Sciences, San Paolo Hospital, University of Milan, 20142 Milano, Italy; E-Mails: aida.zulueta@unimi.it (A.Z.); anna.caretti@unimi.it (A.C.); 3 Department of Experimental, Diagnostic and Specialty Medicine (DIMES), University of Bologna, 40126 Bologna, Italy; E-Mail: fabio.dallolio@unibo.it

**Keywords:** DNA methylation, regulation of gene expression, cancer, glycoconjugates

## Abstract

Glycosylation is a metabolic pathway consisting of the enzymatic modification of proteins and lipids through the stepwise addition of sugars that gives rise to glycoconjugates. To determine the full complement of glycoconjugates that cells produce (the glycome), a variety of genes are involved, many of which are regulated by DNA methylation. The aim of the present review is to briefly describe some relevant examples of glycosylation-related genes whose DNA methylation has been implicated in their regulation and to focus on the intriguing case of a glycosyltransferase gene (*B3GALT5*). Aberrant promoter methylation is frequently at the basis of their modulation in cancer, but in the case of *B3GALT5*, at least two promoters are involved in regulation, and a complex interplay is reported to occur between transcription factors, chromatin remodelling and DNA methylation of typical CpG islands or even of other CpG dinucleotides. Transcription of the *B3GALT5* gene underwent a particular evolutionary fate, so that promoter hypermethylation, acting on one transcript, and hypomethylation of other sequences, acting on the other, cooperate on one gene to obtain full cancer-associated silencing. The findings may also help in unravelling the complex origin of serum CA19.9 antigen circulating in some patients.

## 1. Introduction

The extreme complexity of multicellular organisms requires that their molecular and cellular interactions are tuned with absolute precision in terms of temporal and spatial organization. Compared with that of a simple multicellular organism of few hundred cells only, such as *C. elegans*, the organization of a mammal of several billion cells is more complex by many orders of magnitude. This level of complexity requires that the information contained in the genetic code is translated in molecular and cellular interactions by mechanisms ensuring both fidelity and flexibility. While fidelity is easily guaranteed by the classical deterministic mechanisms at the basis of the template-driven biosynthesis of nucleic acids and proteins, flexibility requires highly-tunable and promptly-reversible mechanisms. Evolution has developed levels of control of gene expression and of protein function that fulfill these requirements: the first is represented by the epigenetic mechanisms regulating transcription; the second by posttranslational modifications of proteins, among which glycosylation plays a pivotal role.

Glycosylation is a metabolic pathway consisting of the enzymatic modification of proteins and lipids through the stepwise addition of sugars that gives rise to the glycoconjugates (or glycans): glycoproteins, glycolipids and proteoglycans. Unlike the biosynthesis of nucleic acids or proteins, which is a deterministic process, glycosylation is a stochastic (or probabilistic) event in which the final sugar chain product results from the cooperative and competitive interaction of multiple players. A pivotal role in glycosylation is played by glycosyltransferases, the enzymes responsible for the step-wise addition of individual sugar residues, but other factors, such as the availability of the sugar donors, of the protein or lipid substrates, or the action of the glycohydrolases, contribute to determine the full complement of glycans that cells produce (the glycome). Historically, protein- or lipid-linked sugar chains have been considered to play multiple biological roles [1], and in general, it can be said that they mediate the “fine tuning” of cellular and molecular interactions. This means that, besides contributing to biological processes through carbohydrate-protein interactions, sugar chains can also regulate protein-protein interactions. An example of a carbohydrate-protein interaction is provided by the cell adhesion phenomena mediated through members of the selectin family and their carbohydrate ligands as the sialyl Lewis antigens [2]. An example of protein-protein interaction finely tuned by the action of carbohydrates is provided by immunoglobulins G, where the sugar chain N-linked to asparagine 297 of their Fc portion determines the pro- or anti-inflammatory effects of the antibody [3].

Epigenetic chromatin modifications and protein or lipid glycosylation, although profoundly different from the mechanistic point of view, share important key features. First of all, they are both the product of non-deterministic processes. DNA methylation and histone methylation/acetylation depend on the relative abundance of the enzymes adding or removing methyl and acetyl groups, and glycosylation depends on that of enzymes adding or removing sugar residues. Second, both processes are reversible, although usually very stable. Third, although both modifications can have a huge functional impact on DNA and proteins, the basic information carried by these macromolecules resides at the level of their primary sequence.

Epigenetic mechanisms deeply impact the glycosylation machinery through either DNA methylation or histone methylation/acetylation, or both (many of them are reviewed in [4] and [5]). Relevant examples include the biosynthesis of bioactive molecules, such as the histo-blood group ABO, Lewis and Sda antigens, the T antigen, the mucins [6] and the GlcNAcylation of proteins, which, in turn, affects the epigenome *per se* [7,8].

Through epigenetic mechanisms of regulation, the genome can cope with environmental challenges without the need to select advantageous random mutations, a process that would require many generations. Analogously, an antibody with a given antigen specificity can change its downstream effects simply by adding or removing a specific monosaccharide on its N-linked chain.

It has been proposed that the epigenetic control of networks of genes, like those involved in glycosylation, is a crucial resource used by higher organisms to compete or collaborate with microorganisms [5] in response to environmental stimuli. Even more interesting, there is increasing evidence of the transgenerational transmission of epigenetic changes [9,10]; however, the molecular mechanisms responsible for the transmission of these changes to gametes remain unknown.

Despite such an enormous possibility of future research, the present state-of-the-art concerning the complex interplay among genetic and epigenetic mechanisms regulating the expression of glycosylation-related genes (glycogenes) is far from being elucidated.

In the last few decades, an innumerable amount of research articles reported the silencing of genes determined by DNA methylation in the context of CpG islands located near promoters and the consequent possibility of reactivating them through action on demethylating agents, with particular focus on cancer-associated gene silencing. This overwhelming stream of data point to the equation: DNA hypermethylation equals cancer equals gene silencing. However, it has been reported since the 1980s [11,12] that cancer transformation is indeed associated with a global DNA hypomethylation, since the majority of CpG dinucleotides, scattered in the genome at a relative low density and far from gene promoters, are indeed hypomethylated in cancer [13,14]. In other words, only CG dinucleotides within DNA regions of high CpG density, *i.e.*, CpG islands frequently located near gene promoters, are hypermethylated in cancer and hypomethylated in normal cells, while the opposite occurs for all other CpGs. While the strong association between CpG island hypermethylation and gene silencing in cancer has been widely documented and characterized for a long time, the role of the hypermethylation of the other CpG dinucleotides in normal cells *versus* hypomethylation in cancer has not been investigated until very recently. An increasing amount of recent data suggests that the methylation of CpG dinucleotides outside typical CpG islands plays a relevant role in alternative promoter usage, regulation of short and non-coding RNAs, alternative RNA processing and enhancer activity, even in the context of neoplastic transformation [15].

The aim of the present review is to briefly describe some relevant examples of glycogenes controlled through DNA methylation and to focus on the intriguing case of a glycosyltransferase gene, the regulation of which involves at least two promoters and a complex interplay between transcription factors, chromatin remodelling, DNA methylation of typical CpG islands or even of other CpG dinucleotides. It appears responsible for efficient cancer-associated silencing and probably fine tissue-specific expression of a galactosyltransferase enzyme isoform (B3GALT5), which also underwent a particular evolutionary fate.

## 2. Methylation Control of Glycogenes

The global effect of methylation on the glycome was studied by high-throughput techniques in cells treated with 5-aza-2’-deoxycytidine (5AZA), a DNA methyltransferase inhibitor that has shown substantial potency in reactivating epigenetically silenced tumor suppressor genes. It has revealed a strong impact on sialylation, core fucosylation and N-linked branching [16]. Other relevant examples are the following.

Glycosyltransferases: A variety of glycosyltransferases are regulated by epigenetic mechanisms, mainly promoter methylation. Aberrant promoter methylation is often at the basis of glycosyltransferase modulation in cancer. Examples are provided by enzymes directly involved in selectin ligand biosynthesis [17], such as sialyltransferase ST3GAL6 [18] and fucosyltransferase FUT3 [19], as well as enzymes affecting indirectly selectin ligand biosynthesis, because of their involvement in the biosynthesis of alternative structures, such as ST6GALNAC6 [20], the sulfate transporter, DTDST [21], or B4GALNT2 [22,23,24]. The latter provides an example of a gene downregulated in cancer, in which promoter demethylation is a condition necessary, but not sufficient to restore a physiological level of enzyme expression [25]. Furthermore, glycosyltransferases involved in the high branching of the N-linked chains of glycoproteins, such as GlcNAcT-IV [26,27] and GlcNAcT-V [28], display methylation-dependent modulation in cancer. Finally, the downregulation of the glycosyltransferase responsible for the biosynthesis of A antigen (of the ABO blood group system), frequently observed in cancer, is at least partially dependent on the hypermethylation of its promoter [29,30,31].

Enzymes of sugar nucleotides biosynthesis: The methylation-dependent inhibition of UDP-GlcNAc 2-epimerase/ManNAc kinase, the key enzyme of the biosynthesis of the sugar donor, CMP-sialic acid, may be responsible for decreased sialylation, even in the presence of unaltered sialyltransferase levels [32,33]. This modification can be induced by latent HIV infection of T-cells [32].

Galectins: Galectins are galactose-specific mammalian lectins that mediate a variety of biological phenomena, including cell proliferation and apoptosis, either through binding to galactose-containing glycoconjugates or through carbohydrate-independent intracellular mechanisms [34]. Promoter methylation appears to be the major mechanism regulating galectin expression in physiological and pathological conditions. Galectin-1, a product of the *LGALS1* locus, induces apoptosis of T-lymphocytes and cancer cells. In colorectal cancer, galectin-1 expression is silenced by promoter hypermethylation, resulting in reduced apoptosis [35], while in mixed lineage leukemia (*MLL*)-rearranged B-lymphoblastic leukemias, galectin-1 is overexpressed, because of the histone methylation of the LGALS1 promoter [36]. Galectin-3 expression is frequently altered in cancers with divergent effects on tumor growth [37]. In prostate tissues, the galectin-3 promoter is unmethylated, while it becomes strongly methylated in the early stages of prostate cancer, but less methylated in high-grade cancers [38,39]. Galectin-3 modulation is controlled by promoter methylation also in thyroid cancer [40], in colon cancer of the mucinous type [41] and in pituitary tumors [42]. Galectin-7 acts as a tumor suppressor in gastric cancer, and its downregulation is due to promoter hypermethylation [43], while in lymphoma progression, it is upregulated, because of promoter hypo-methylation [44].

## 3. The Intriguing Case of the *B3GALT5* Gene

**Figure 1:**
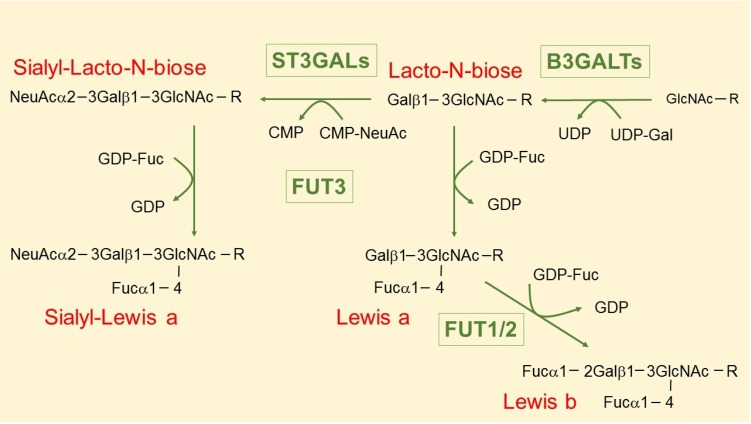
Biosynthesis of type 1 chain Lewis antigens. B3GALTs include B3GALT5, B3GALT1 and B3GALT2; ST3GALs are α2,3 sialyltransferases acting on galactose and include ST3GAL3 and possibly ST3GAL4; FUT3 is a unique α1,4 fucosyltransferase acting on N-acetylglucosamine; FUT1 and FUT2 are α1,2 fucosyltransferases acting on galactose.

β1,3 galactosyltransferase B3GALT5 is responsible for type 1 chain oligosaccharide synthesis, including the selectin ligand, sialyl-Lewis a, epitope of tumor marker CA19.9 and other Lewis antigens, such as Lewis a and Lewis b (Figure 1) [45,46].

**Figure 2:**
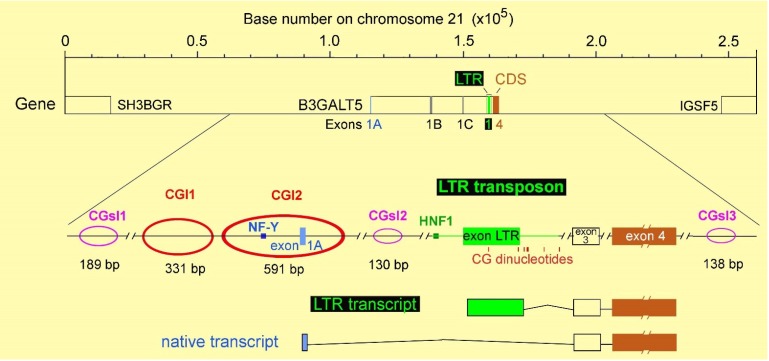
Genomic organization and DNA methylation of the *B3GALT5* gene. In the upper part, the genomic sequence of chromosome 21 (0.26 Mbp) encompassing *B3GALT5* and intergenic regions is presented. The scale starts at 40.87 Mbp and ends at 41.13 Mbp of NCBI Reference Sequence: NC_000021.8. The LTR transposon starts at bp 41029095 of such a sequence. Between alternative 5'UTRs, exons 1A and 1 (LTR) are the most relevant in various tissues. In the lower part, about 50 Kbp were enlarged to show transcription factor binding sites, CpG islands (CGI, in red), CpG short islands (CGsI) of at least 100 bp (pink), CG dinucleotides present in the LTR transposon and the two main transcripts.

In mammary gland, thymus and trachea, as well as in some human cancer cell lines, transcription is mainly driven by a promoter that was found to be sensitive to nuclear factor NF-Y also in mice [47]. Due to the conservation among distant mammalian species, it was referred to as the native promoter. In the organs of the gastrointestinal tract (such as the colon, stomach and pancreas) acts another promoter, stronger than the native promoter [47,48,49]. This alternative promoter has a retroviral origin (named LTR), which was probably acquired about 25–30 million years ago [50] and is regulated through the hepatocyte nuclear factor HNF1 [48,49]. In various cell lines of different tissue origin, but even among those derived from the same tissue, B3GALT5 transcript, as a whole, is differentially expressed [45,51]. Moreover, it is strongly downregulated in colon cancer with respect to the normal mucosa [48,52]. Surprisingly, the amounts of transcription factors involved in the regulation of either transcript were not correlated with the expression levels of the cognate transcript. In particular, NF-Y is rather ubiquitous [53] and well detected in several cell lines, where the native B3GALT5 transcript is completely absent, or in colon cancer biopsies, where it is faintly detected [54]. Similarly, HNF1α or β, or both, are also easily detected in cell lines or tissues, including colon cancers, where the levels of the B3GALT5 LTR transcript are minimal or undetectable, opening the question about the mechanisms responsible for tissue-specific expression and cancer downregulation of both transcripts [55]. Interestingly, the native promoter is located between two CpG islands, while the 650 bp-long LTR transposon, which includes a shorter LTR first exon and the cognate promoter, contains only seven dispersed CG pairs, and no CpG island is present in the proximal sequences (Figure 2).

## 4. Regulation of *B3GALT5* Native Promoter

Methylation analysis of CpG islands 1 and 2 surrounding the native promoter, performed by both quantitative pyrosequencing and direct bisulfite sequencing of 12 and 66 CG dinucleotides of CpG islands 1 and 2, respectively, indicated a strong inverse correlation between transcript expression and DNA methylation status [54]. In particular, in HuCC-T1 cells, which express the highest level of the transcript, CpG 2 appeared almost unmethylated, and CpG island 1 was just scarcely methylated (mean methylation levels ~20%). In MKN-45 and MCF-7 cell lines, where the expression levels of transcript are from low to moderate, CpG island 1 was more methylated (mean methylation values ~80% in both lines) while CpG island 2 was almost unmethylated in MCF-7 and mildly methylated in MKN-45 (mean methylation levels ~30%). In HCT-15 and MDA-MB-231 cells, where the transcript is undetectable, both CpG islands were hypermethylated (form 70% to 90%). In matched normal and tumor colon samples, the methylation levels of both islands were increased in cancer with respect to the corresponding normal mucosa. In the latter, where the expression of the transcript is detectable at different levels, mean methylation values of CpG island 1 were below 50%, and those of CpG island 2 below 20%. In cancer samples, this increases up to 60%–70% in CpG island 1 and up to 40% in CpG island 2. Similarly, the degree of methylation was higher in breast cancer biopsies than in normal counterparts, suggesting that DNA methylation of the *B3GALT5* native promoter probably accounts for transcript silencing in cancer.

The quantitative ChIP assay from the above-mentioned cell lines expressing different levels of B3GALT5 native transcript confirmed that the transcriptional activity of the native promoter is associated with chromatin status. In fact, high expression of the transcript (HuCC-T1 cells) was found together with high levels of modifications associated with transcriptionally competent chromatin (H3K4me3, H3K79me2, H3K9Ac and H3K9-14Ac) and low levels of those related to silenced chromatin (H3K27me2 and H4K20me3). Moderate to low expression of the transcript (MCF-7 and MKN-45 cells) was associated with a similar pattern, with quantitative differences especially in MKN-45 cells. The absence of the transcript (HCT-15 and MDA-MB-231 cells) was related to the opposite histone code. These cell lines, in fact, resulted in being negative for H3K4me3, H3K79me2 and H3K9-14Ac and positive for H3K27me2, H4K20me3 and H3K9Ac.

Treatment of such cells for different times with different concentrations of DNA methyltransferase inhibitor 5AZA or histone deacetylase inhibitor Trichostatin A (TSA), or with a combination of both, was unable to restore a detectable expression. However, the treatment was able to reduce methylation from ~80% to ~60% in MDA-MB-231 and from ~70% to ~40% in HCT-15. Indeed, both agents were able to increase the expression of the transcript in MKN-45 cells that express a low amount *per se* of the transcript. In particular, either TSA or 5AZA treatments increased the expression by ~80%. A combination of both drugs failed to provide any further improvement. In line with expression finding, pyrosequencing analysis of MKN-45 cells treated with 5AZA showed demethylation of both islands. Altogether, the results suggest that complex epigenetic modulation underlies the regulation of the native *B3GALT5* promoter (Table 1).

**Table 1:** Epigenetic features affecting B3GALT5 transcripts in various cell lines.

	HuCC-T1	COLO-205	HCT-15	MKN-45	MCF-7	MDA-MB-231
**NATIVE TRANSCRIPT**						
**Expression levels**	++	++	-	+	+	-
**Effect of 5AZA**	=	ND	=	↑	ND	=
**Effect of TSA**	=	ND	=	↑	ND	=
**Methylation status of CpG island 1/2 (%)**	20/5	ND	80/75	80/30	80/5	90/80
**H3K27me2, H4K20me3**	very low	ND	high	low	low	high
**H3K4me3, H3K79me2, H3K9Ac, H3K9-14Ac**	very high	ND	low	high	high	low
**LTR TRANSCRIPT**						
**Expression levels**	-	++++	-	++	-	-
**Effect of 5AZA**	=	↓↓	=	↓↓	ND	ND
**Effect of TSA**	=	=	=	=	ND	ND

ND: not determined. TSA, Trichostatin A.

## 5. Regulation of the *B3GALT5* LTR Promoter

In MKN-45 cells, the LTR promoter of *B3GALT5* is also expressed, and the amount under steady-state conditions is about eight-times higher than that of the native transcript. Surprisingly, the sensitivity to the same drugs was totally different: TSA treatment had no effect on the LTR transcript, while 5AZA strongly impaired expression. Treatment with 5AZA of COLO-205 cells, which express the LTR transcript at the highest levels found, provided similar results: strong silencing of the B3GALT5 LTR transcript and no effect on HNF1. Interestingly, LTR promoter analysis *in vitro* indicated that the HNF1 binding site is the only functional part of the LTR promoter, and that no other binding sites, for stimulatory or inhibitory factors, are physiologically relevant [55]. Conversely, DNA demethylation obtained through 5AZA treatment reproduced *in vitro* the downregulation of the transcript observed among cell lines and cancer biopsies *in vivo.* In fact, in treated cells, the levels of the B3GALT5 LTR transcript decreased from 3–10 to less than 0.2 fg/pg β-actin, while the amounts of HNF1 remained unchanged, as found in colon cancer biopsies. Since LTR and proximal sequences do not contain CpG islands, the methylation-sensitive DNA sequences probably represent element(s) involved in transcriptional regulation residing outside the LTR sequence and distant from the promoter. Alignment of the LTR sequence and the whole *B3GALT5* gene in the context of chromosome 21 (Figure 2) revealed that the CpG islands regulating transcription of the native B3GALT5 mRNA are the only typical promoter-associated CpG islands present. However, shorter stretches of CpG dinucleotides, referred to as CpG short islands, were detected using the EMBOSS Cpgplot software, as reported [55]. They were found one in an intron, and the others in the intergenic regions [55]. Unfortunately, due to the extremely high homology of this human sequence with that of the other primates sharing the LTR transposon [50], no prediction can be made *in silico* about the relevance of any such islands.

The methylation of stretches of CpG dinucleotides shorter than CpG islands associated with promoters is emerging as a relevant aspect of transcriptional control [15], being responsible for the recruitment of alternative promoters, regulation of non-coding RNA synthesis or modulation of enhancer activity. In particular, hypomethylation of enhancer sequences is reported to negatively regulate transcription in cancer and during tissue differentiation [15]. The occurrence of distal regulatory elements binding transcription factors in a methylation-dependent manner was recently reported even in breast cancer [56]. *B3GALT5* transcription thus represents a promising model to address such novel issues, since hypomethylation of distant sequences, acting on the LTR transcript, and promoter hypermethylation, acting on the native transcript [54,55], cooperate on one gene to obtain full cancer-associated silencing.

Since the two transcripts differ in their 5'UTRs only, the involvement of a common non-coding RNA in the regulation of both can be hypothesized. However, since the effect of demethylating agents on the two transcripts is almost the opposite, the existence of a regulatory 3' CpG island sequence appears not probable. On the other hand, the involvement of transcriptional activators requiring the methylation of specific CG dinucleotides for efficient binding can be postulated. In this regard, we have started performing bisulfite sequencing of stretches of CG dinucleotides located at various distances from the LTR promoter. Preliminary results in cancer cell lines suggest an association between the methylation of specific CG dinucleotides and the expression levels of the LTR transcript, thus supporting such a working hypothesis.

The fine methylation-dependent silencing of *B3GALT5* reported in colon cancer has the potential to represent a wider cancer-associated phenomenon, suggesting that many cancers, including those arising in the pancreas or stomach, may lack the expression of B3GALT5 transcripts and cognate Lewis antigens. In light of very recent findings demonstrating that the CA19.9 detected by immunohistochemistry in cancer specimens seems to be a technical artifact [57], the problem of the origin of CA19.9 circulating in cancer patient sera appears very relevant due to the clinical implications. In fact, the assessment of the actual CA19.9 status of a colon cancer may be very important for the prognosis, since the expression of the true antigen promotes angiogenesis and, in turn, tumor growth [57]. On the other side, recent findings indicate that the value of serum CA19.9 is not able to predict the actual expression of the antigen by colon cancer cells, as it has been assumed so far [58]. Our working hypothesis involves the possibility that mainly normal epithelial cells of gastrointestinal origin express B3GALT5 and cognate type 1 chain Lewis antigens, due to the strong epigenetic constrains. Metabolic pathways of such cells may be deranged as a consequence of the invading tumor mass, resulting in an increased reabsorption of the CA19.9 antigen in the blood stream. Interestingly, in cells of different histological origin as those of the prostate, it was recently reported that a defined CA19.9 molecule, where the sLea antigen is carried by MUC1 mucin, a different β1,3 galactosyltransferase isoenzyme, B3GALT1, is specifically involved in the biosynthesis, which appears upregulated by the enhancement of acetylated histone-3 and histone-4 induced by suberoylanilide hydroxamic acid [59].

## 6. Conclusions

The role of DNA methylation has been restricted for several years to CpGs belonging to islands located nearby gene promoters, with particular emphasis on their hypermethylated status associated with gene silencing in cancer and on the possibility to restore expression using demethylating agents. The *B3GALT5* gene offers an intriguing example of the role of distant DNA sequences in controlling a promoter of retroviral origin, where CpG dinucleotides are probably dispersed or assembled in non-canonical islands. In this case, demethylation determines gene silencing and is associated with cancer. Concurrently, another promoter of the same gene is instead affected by the methylation status of classic CpG islands, but its activity is poorly restored by demethylating agents. Noteworthy is that the transcript levels correlate with enzyme activity and, in turn, substrate glycosylation in the case of B3GALT5 (51), while this is not yet known for some other epigenetically-regulated glycogenes. Future studies are necessary to directly show the link between epigenetic regulation of transcription and the actual effect on the cell glycosylation pattern.

## References

[1] Varki A. (1993). Biological roles of oligosaccharides: All of the theories are correct. Glycobiology.

[2] Chase S.D., Magnani J.L., Simon S.I. (2012). E-selectin ligands as mechano sensitive receptors on neutrophils in health and disease. Ann. Biomed. Eng..

[3] Dall’Olio F., Vanhooren C.V., Chen C.P., Slagboom E., Wuhrer M., Franceschi C. (2013). N-glycomic biomarkers of biological aging and longevity: A link with inflammaging. Ageing Res. Rev..

[4] Zoldoš V., Novokmet M., Bečeheli I., Lauc G. (2013). Genomics and epigenomics of the human glycome. Glycoconj. J..

[5] Lauc G., Vojta A., Zoldoš V. (2014). Epigenetic regulation of glycosylation is the quantum mechanics of biology. Biochim. Biophys. Acta.

[6] Yamada N., Kitamoto S., Yokoyama S., Hamada T., Goto M., Tsutsumida H., Higashi M., Yonezawa S. (2011). Epigenetic regulation of mucin genes in human cancers. Clin. Epigenet..

[7] McLarty J.L., Marsh S.A., Chatham J.C. (2013). Post-translational protein modification by O-linked N-acetyl-glucosamine: Its role in mediating the adverse effects of diabetes on the heart. Life Sci..

[8] Hanover J.A., Krause M.W., Love D.C. (2012). Bittersweet memories: Linking metabolism to epigenetics through O-GlcNAcylation. Nat. Rev. Mol. Cell. Biol..

[9] Jablonka E., Raz G. (2009). Transgenerational epigenetic inheritance: Prevalence, mechanisms, and implications for the study of heredity and evolution. Q. Rev. Biol..

[10] Schmitz R.J., Schultz M.D., Lewsey M.G., O’Malley R.C., Urich M.A., Libiger O., Schork N.J., Ecker J.R. (2011). Transgenerational epigenetic instability is a source of novel methylation variants. Science.

[11] Gama-Sosa M.A., Slagel V.A., Trewyn R.W., Oxenhandler R., Kuo K.C., Gehrke C.W., Ehrlich M. (1983). The 5-methylcytosine content of DNA from human tumors. Nucleic Acids Res..

[12] Feinberg A.P., Gehrke C.W., Kuo K.C., Ehrlich M. (1988). Reduced genomic 5-methylcytosine content in human colonic neoplasia. Cancer Res..

[13] Ehrlich M. (2002). DNA methylation in cancer: Too much, but also too little. Oncogene.

[14] Ehrlich M. (2009). DNA hypomethylation in cancer cells. Epigenomics.

[15] Kulis M., Queirós A.C., Beekman R., Martín-Subero J.I. (2013). Intragenic DNA methylation in transcriptional regulation, normal differentiation and cancer. Biochim. Biophys. Acta.

[16] Saldova R., Dempsey E., Perez-Garay M., Marino K., Watson J.A., Blanco-Fernandez A., Struwe W.B., Harvey D.J., Madden S.F., Peracaula R. (2011). 5-AZA-2'-deoxycytidine induced demethylation influences N-glycosylation of secreted glycoproteins in ovarian cancer. Epigenetics.

[17] Syrbe U., Jennrich S., Schottelius A., Richter A., Radbruch A., Hamann A. (2004). Differential regulation of P-selectin ligand expression in naive *versus* memory CD4+ T cells: Evidence for epigenetic regulation of involved glycosyltransferase genes. Blood.

[18] Chachadi V.B., Cheng H., Klinkebiel D., Christman J.K., Cheng P.W. (2011). 5-Aza-2'-deoxycytidine increases sialyl Lewis X on MUC1 by stimulating β-galactoside: α2,3-sialyltransferase 6 gene. Int. J. Biochem. Cell Biol..

[19] Serpa J., Mesquita P., Mendes N., Oliveira C., Almeida R., Santos-Silva F., Reis C.A., Lependu J., David L. (2006). Expression of Le^a^ in gastric cancer cell lines depends on FUT3 expression regulated by promoter methylation. Cancer Lett..

[20] Miyazaki K., Ohmori K., Izawa M., Koike T., Kumamoto K., Furukawa K., Ando T., Kiso M., Yamaji T., Hashimoto Y. (2004). Loss of disialyl Lewis^a^ the ligand for lymphocyte inhibitory receptor sialic acid-binding immunoglobulin-like lectin-7 (Siglec-7) associated with increased sialyl Lewis^a^ expression on human colon cancers. Cancer Res..

[21] Yusa A., Miyazaki K., Kimura N., Izawa M., Kannagi R. (2010). Epigenetic silencing of the sulfate transporter gene DTDST induces sialyl Lewisx expression and accelerates proliferation of colon cancer cells. Cancer Res..

[22] Kawamura Y.I., Toyota M., Kawashima R., Hagiwara T., Suzuki H., Imai K., Shinomura Y., Tokino T., Kannagi R., Dohi T. (2008). DNA hypermethylation contributes to incomplete synthesis of carbohydrate determinants in gastrointestinal cancer. Gastroenterology.

[23] Tong W.G., Wierda W.G., Lin E., Kuang S.Q., Bekele B.N., Estrov Z., Wei Y., Yang H., Keating M.J., Garcia-Manero G. (2010). Genome-wide DNA methylation profiling of chronic lymphocytic leukemia allows identification of epigenetically repressed molecular pathways with clinical impact. Epigenetics.

[24] Wang H.R., Hsieh C.Y., Twu Y.C., Yu L.C. (2008). Expression of the human Sd^a^ β-1,4-N-acetylgalactosaminyltransferase II gene is dependent on the promoter methylation status. Glycobiology.

[25] Dall’Olio F., Malagolini N., Chiricolo M., Trinchera M., Harduin-Lepers A. (2014). The expanding roles of the Sd^a^/Cad carbohydrate antigen and its cognate glycosyltransferase B4GALNT2. Biochim. Biophys. Acta.

[26] Ide Y., Miyoshi E., Nakagawa T., Gu J., Tanemura M., Nishida T., Ito T., Yamamoto H., Kozutsumi Y., Taniguchi N. (2006). Aberrant expression of N-acetylglucosaminyltransferase-IVa and IVb (GnT-IVa and b) in pancreatic cancer. Biochem. Biophys. Res. Commun..

[27] Kizuka Y., Kitazume S., Yoshida M., Taniguchi N. (2011). Brain-specific expression of N-acetylglucosaminyltransferase IX (GnT-IX) is regulated by epigenetic histone modifications. J. Biol. Chem..

[28] Chakraborty A.K., Sousa J.F., Chakraborty D., Funasaka Y., Bhattacharya M., Chatterjee A., Pawelek J. (2006). GnT-V expression and metastatic phenotypes in macrophage-melanoma fusion hybrids is down-regulated by 5-Aza-dC: Evidence for methylation sensitive, extragenic regulation of GnT-V transcription. Gene.

[29] Chihara Y., Sugano K., Kobayashi A., Kanai Y., Yamamoto H., Nakazono M., Fujimoto H., Kakizoe T., Fujimoto K., Hirohashi S. (2005). Loss of blood group A antigen expression in bladder cancer caused by allelic loss and/or methylation of the ABO gene. Lab. Invest..

[30] Dabelsteen E., Gao S. (2005). ABO blood-group antigens in oral cancer. J. Dent. Res..

[31] Gao S., Worm J., Guldberg P., Eiberg H., Krogdahl A., Liu C.J., Reibel J., Dabelsteen E. (2004). Genetic and epigenetic alterations of the blood group ABO gene in oral squamous cell carcinoma. Int. J. Cancer.

[32] Giordanengo V., Ollier L., Lanteri M., Lesimple J., March D., Thyss S., Lefebvre J.C. (2004). Epigenetic reprogramming of UDP-N-acetylglucosamine 2-epimerase/N-acetylmannosamine kinase (GNE) in HIV-1-infected CEM T cells. FASEB J..

[33] Oetke C., Hinderlich S., Reutter W., Pawlita M. (2003). Epigenetically mediated loss of UDP-GlcNAc 2-epimerase/ManNAc kinase expression in hyposialylated cell lines. Biochem. Biophys. Res. Commun..

[34] Boscher C., Dennis J.W., Nabi I.R. (2011). Glycosylation, galectins and cellular signaling. Curr. Opin. Cell Biol..

[35] Satelli A., Rao U.S. (2011). Galectin-1 is silenced by promoter hypermethylation and its re-expression induces apoptosis in human colorectal cancer cells. Cancer Lett..

[36] Juszczynski P., Rodig S.J., Ouyang J., O’Donnell E., Takeyama K., Mlynarski W., Mycko K., Szczepanski T., Gaworczyk A., Krivtsov A. (2010). MLL-rearranged B lymphoblastic leukemias selectively express the immunoregulatory carbohydrate-binding protein galectin-1. Clin. Cancer Res..

[37] Newlaczyl A.U., Yu L.G. (2011). Galectin-3—A jack-of-all-trades in cancer. Cancer Lett..

[38] Ahmed H., Banerjee P.P., Vasta G.R. (2007). Differential expression of galectins in normal, benign and malignant prostate epithelial cells: Silencing of galectin-3 expression in prostate cancer by its promoter methylation. Biochem. Biophys. Res. Commun..

[39] Ahmed H., Cappello F., Rodolico V., Vasta G.R. (2009). Evidence of heavy methylation in the galectin 3 promoter in early stages of prostate adenocarcinoma: Development and validation of a methylated marker for early diagnosis of prostate cancer. Transl. Oncol..

[40] Keller S., Angrisano T., Florio E., Pero R., Decaussin-Petrucci M., Troncone G., Capasso M., Lembo F., Fusco A., Chiariotti L. (2013). DNA methylation state of the galectin-3 gene represents a potential new marker of thyroid malignancy. Oncol. Lett..

[41] Ben Mahmoud L.K., Arfaoui A., Khiari M., Chaar I., El Amine O., Ben Hmida A.M., Gharbi L., Mzabi S.R., Bouraoui S. (2011). Loss of galectin-3 expression in mucinous colorectal carcinomas is associated with 5’CpG island methylation in Tunisian patients. Appl. Immunohistochem. Mol. Morphol..

[42] Ruebel K.H., Jin L., Qian X., Scheithauer B.W., Kovacs K., Nakamura N., Zhang H., Raz A., Lloyd R.V. (2005). Effects of DNA methylation on galectin-3 expression in pituitary tumors. Cancer Res..

[43] Kim S.J., Hwang J.A., Ro J.Y., Lee Y.S., Chun K.H. (2013). Galectin-7 is epigenetically-regulated tumor suppressor in gastric cancer. Oncotarget.

[44] Demers M., Couillard J., Giglia-Mari G., Magnaldo T., St Pierre Y. (2009). Increased galectin-7 gene expression in lymphoma cells is under the control of DNA methylation. Biochem. Biophys. Res. Commun..

[45] Isshiki S., Kudo T., Nishihara S., Ikehara Y., Togayachi A., Furuya A., Shitara K., Kubota T., Watanabe M., Kitajima M. (1999). Cloning, expression, and characterization of a novel UDP-galactose: β-*N*-acetylglucosamine β1,3-galactosyltransferase (β3Gal-T5) responsible for synthesis of type 1 chain in colorectal and pancreatic epithelia and tumor cells derived therefrom. J. Biol. Chem..

[46] Lin C.H., Fan Y.Y., Chen Y.Y., Wang S.H., Chen C.I., Yu L.C., Khoo K.H. (2009). Enhanced expression of beta 3-galactosyltransferase 5 activity is sufficient to induce *in vivo* synthesis of extended type 1 chains on lactosylceramides of selected human colonic carcinoma cell lines. Glycobiology.

[47] Mare L., Trinchera M. (2007). Comparative analysis of retroviral and native promoters driving expression of beta1,3-galactosyltransferase beta3Gal-T5 in human and mouse tissues. J. Biol. Chem..

[48] Isshiki S., Togayachi A., Kudo T., Nishihara S., Watanabe M., Kubota T., Kitajima M., Shiraishi N., Sasaki K., Andoh T. (2003). Lewis type 1 antigen synthase (beta3Gal-T5) is transcriptionally regulated by homeoproteins. J. Biol. Chem..

[49] Dunn C.A., Medstrand P., Mager D.L. (2003). An endogenous retroviral long terminal repeat is the dominant promoter for human beta1,3-galactosyltransferase 5 in the colon. Proc. Natl. Acad. Sci. USA.

[50] Dunn C.A., van de Lagemaat L.N., Baillie G.J., Mager D.L. (2005). Endogenous retrovirus long terminal repeats as ready-to-use mobile promoters: The case of primate beta3GAL-T5. Gene.

[51] Mare L., Trinchera M. (2004). Suppression of β1,3galactosyltransferase β3Gal-T5 in cancer cells reduces sialyl-Lewis a and enhances poly N-acetyllactosamines and sialyl-Lewis x on O-glycans. Eur. J. Biochem..

[52] Salvini R., Bardoni A., Valli M., Trinchera M. (2001). Beta 1,3-Galactosyltransferase beta 3Gal-T5 acts on the GlcNAcbeta 1-->3Galbeta 1-->4GlcNAcbeta 1-->R sugar chains of carcinoembryonic antigen and other N-linked glycoproteins and is down-regulated in colon adenocarcinomas. J. Biol. Chem..

[53] Caretti G., Salsi V., Vecchi C., Imbriano C., Mantovani R. (2003). Dynamic recruitment of NF-Y and histone acetyltransferases on cell-cycle promoters. J. Biol. Chem..

[54] Caretti A., Sirchia S.M., Tabano S., Zulueta A., Dall’Olio F., Trinchera M. (2012). DNA methylation and histone modifications modulate the β1,3 galactosyltransferase β3Gal-T5 native promoter in cancer cells. Int. J. Biochem. Cell. Biol..

[55] Zulueta A., Caretti A., Signorelli P., Dall’olio F., Trinchera M. (2014). Transcriptional control of the *B3GALT5* gene by a retroviral promoter and methylation of distant regulatory elements. FASEB J..

[56] Aran D., Sabato S., Hellman A. (2013). DNA methylation of distal regulatory sites characterizes dysregulation of cancer genes. Genome Biol..

[57] Terraneo L., Avagliano L., Caretti A., Bianciardi P., Tosi D., Bulfamante G.P., Samaja M., Trinchera M. (2013). Expression of carbohydrate-antigen sialyl-Lewis a on colon cancer cells promotes xenograft growth and angiogenesis in nude mice. Int. J. Biochem. Cell. Biol..

[58] Mare L., Caretti A., Albertini R., Trinchera M. (2013). CA19.9 antigen circulating in the serum of colon cancer patients: Where is it from?. Int. J. Biochem. Cell. Biol..

[59] Chachadi V.B., Ali M.F., Cheng P.W. (2013). Prostatic cell-specific regulation of the synthesis of MUC1-associated sialyl Lewis a. PLoS One.

